# A Modified Calculation of the Withdrawal Time and a Risk Assessment of Enrofloxacin in *Micropterus salmoides* after Its Ad Libitum Administration via Medicated Feed in the Commercial Aquaculture

**DOI:** 10.3390/ani14162341

**Published:** 2024-08-14

**Authors:** Ning Xu, Yongzhen Ding, Xiaohui Ai

**Affiliations:** 1 Yangtze River Fisheries Research Institute, Chinese Academy of Fishery Sciences, Wuhan 430223, China; xuning@yfi.ac.cn; 2 Agro-Environmental Protection Institute, Ministry of Agriculture and Rural Affairs, Tianjin 300191, China

**Keywords:** enrofloxacin, withdrawal time, medicated feed, statistical approaches, largemouth bass

## Abstract

**Simple Summary:**

In commercial aquaculture, aquatic animals are generally administered medicine ad libitum (at one’s pleasure) via medicated feed. However, the estimation of withdrawal times for aquatic medicine is mostly carried out through the forced administration of medicine via oral gavage, which is not consistent with commercial aquaculture. This process may influence the absorption, distribution, metabolism, and excretion of medicine in aquatic animals and further affect the calculation of withdrawal times (WTs) for medicines. Therefore, in the current study, we selected the important aquatic medicine enrofloxacin (EF) to estimate its WTs in largemouth bass (*Micropterus salmoides*) after the administration of medicated feed. This study will obtain practical estimations of the WT of EF in actual aquaculture to aid in the surveillance of medication residue in fish products.

**Abstract:**

The present study investigated the residue depletion and WTs of EF and its main metabolite, ciprofloxacin, in largemouth bass after ad libitum administration in commercial fish farming based on statistical approaches. Samples collected at pre-determined time points were assessed using high-performance liquid chromatography. If the concentrations of medicine were less than the quantitative limit, they were set to be half of the limit of quantitative. The terminal elimination of the target compound was assumed to fit a one-compartment model. The statistical methods of Bartlett’s test and Cochran’s test were used to inspect the homogeneity of the log-transformed data. The lack-of-fit test and F-test were used to check the linearity of the regression line. Outliers were assessed using standardized residuals. The final WT was estimated using the 95% percentile with a 95% confidence level. The WTs of EF were calculated to be 46, 29, 33, 46, and 20 days for the muscle + skin, plasma, gill, kidney, and liver, respectively. After the risk assessment, the values of the hazard quotient were calculated to be far less than 1, indicating that the risk of residual EF was particularly low in the edible tissues of largemouth bass after medicine depletion for various WTs.

## 1. Introduction

To date, most of the current withdrawal times (WTs) of aquatic medicines have been evaluated through the administration of medicine via multiple oral gavages under laboratory conditions [1,2,3,4]. This is an average method for administering medicine to aquatic animals, but it easily results in fish stress, leading to death because of repeated catching and fixing during the process of oral gavage. Compared to the method of administering medicine via medicated feed in commercial fish farming, oral gavage makes all medicines a bolus dose, entering the fish body and prolonging WTs. Therefore, if we want to obtain practically meaningful WTs in the tissues of aquatic animals, the method of medicine administration must be consistent with that used in commercial aquaculture.

The largemouth bass (*Micropterus salmoides*) derives from North America. Due to its excellent growth performance and the quality of its meat, the largemouth bass has been acknowledged as a high-quality cultured species, which has been introduced to China, France, Britain, Brazil, South Africa, the Philippines, and other countries. China introduced the largemouth bass in 1983, and artificial reproduction was completed in 1985. So far, the culturing of largemouth bass has become a large industry in China, with an annual production of 814,765 tons [5]. However, this species is easily infected by bacterial pathogens of *Aeromonas* spp., *Nocardia* spp., *Flavobacterium columna*, *Edwardsia* spp., *Vibro anguillarum*, and *Burkholderia cepacian* in high-density environments [6,7,8]. These infections lead to rotten gills, enteritis, and hemorrhagic diseases. Published reports have proved that enrofloxacin (EF) is sensitive to these bacterial pathogens [8,9]. However, the residues of EF (its residue marker is the parent drug and its metabolite ciprofloxacin (CF)) in edible tissues often exceed the maximum residue level (MRL) in commercial aquaculture, which can threaten human health [10]. Hence, the WTs of EF must be re-evaluated in largemouth bass, adopting an identical method of medicine administration to that used in commercial aquaculture.

To establish a reliable WT, risk-assessment methods have been used to verify the safety of residual concentrations after medicine depletion to calculate the WT. In the food safety field, risk assessments of compounds commonly use the indices of the estimated daily intake (EDI) and hazard quotient (HQ) [11]. The EDI indicates the value of daily exposure to the consumption of chemical residue or a nutrient based on the average body weight of humans. The HQ is a parameter based on the EDI divided by the acceptable daily intake (ADI) of the target compound. If the HQ is less than 1, it is considered to be safe for humans. If the HQ is more than 1, it indicates a risk to human health. To date, some studies have carried out a risk assessment of EF in aquatic animals. Hua et al. [12] assessed the residues of 65 antibiotics in 10 kinds of freshwater fish in Southeast China in terms of the health risks for humans. EF was detectable in 50.4% of samples and had a higher level in benthic predatory fish than other fish species. Additionally, EF also possessed the highest concentration and detected rate in the residue screening of 160 fish products from Shandong Province in China [13]. The calculated EDIs of grass carp, carp, and crucian carp were more than 10% of the ADIs for the high dairy consumption group, suggesting that a heavy intake of these fish species may result in potential health risks. Hence, the risk assessment of drug residue in fish products facilitates the establishment of a reliable WT for target compounds in fish species to ensure food safety.

The calculation of the WT of EF has been carried out in channel catfish (*Ictalurus punetaus*) [14], common carp (*Cyprinus carpio*) [15], yellow river carp (*Cyprinus carpio haematoperus*) [16], *Russian sturgeon* (*Acipenser gueldenstaedtii*) [17], crucian carp (*Carassius auratus gibelio*) [18], the pearl gentian grouper (*Epinephelus fuscoguttatus* × *Epinephelus lanceolatus*) [19], *Nile tilapia* (*Oreochromis niloticus*) [20], the northern snakehead (*Channa argus*) [3], Turbot (*Scophthalmus maximus*) [21,22], flounder (*Paralichthys olivaceus*) [22,23], hemiglossus (*Cynoglossus semilaevis*) [22], and crayfish (*Procambarus clarkii*) [24]. However, the method of medicine administration in these studies is oral gavage. If we use this method to establish the WT, it may not be suitable for commercial fish farming. Therefore, this study will implement the assessment of WTs for EF in largemouth bass after multiple oral doses via medicated feed based on the calculation of statistical approaches. Afterwards, a dietary risk assessment was performed using HQs and EDIs to verify the efficacy of the WT. These practical WTs will be beneficial to guiding the culture and fishing of largemouth bass to guarantee human health.

## 2. Materials and Methods

### 2.1. Chemicals and Reagents

Reagents of formic acid, acetonitrile, hydrochloric acid, methanol, and water were all high-performance liquid chromatography (HPLC)-grade and purchased from Thermo Fisher (Waltham, MA, USA). The anhydrous magnesium sulfate, various centrifugal tubes, and vials were purchased from Shanghai CNW Technologies (Shanghai, China). Standards of enrofloxacin (EF, purity ≥ 99.2%) and ciprofloxacin (CF, purity ≥ 95.0%) were bought from Dr. Ehrenstorfer GmbH. (Augsburg, Germany). The commercial powder of EF (purity ≥ 98.0%) was bought from Shandong Lu Kang Animal Medicine Co., Ltd. (Jining, China).

### 2.2. Medicated Feed

A medicine-free pelleted feed consisting of 60.0% fish meal, 10.0% wheat bran, 25.0% corn flour, and 5.0% additives such as yeast, vitamins, and minerals was purchased from Hengyuan Formulation Feed Co., Ltd. (Wuhan, China). Firstly, 3 kg of pelleted feed was smashed using a pulverizer, and excess water was evaporated at 40 °C. After drying for 24 h using an oven, 2.992 kg of smashed feed was put in a tray. Next, 8.0 g of EF was weighed and dissolved in 200 mL of pure water. The EF solution was homogeneously mixed with dried feed to make pellets using a granulation machine. Next, the pellets containing EF were desiccated for 12 h at 40 °C and stored in a waterproof bag to protect them from moisture. Finally, the concentration of EF was detected to be 3982 mg/kg using HPLC.

### 2.3. Animal Rearing and Sampling 

Two hundred largemouth bass (243.6 ± 53.3 g, 12 months old, mixed gender) were obtained from the culturing facility of the College of Fisheries, Huazhong Agricultural University. All fish from the juvenile stage were held in a cylindrical captive barrel (diameter 4 m × height 3 m) with an automatic cleaning system. Dissolved oxygen levels of cultured water remained around 7.0–8.5 mg/L using a microporous aeration tube connected to an aeration pump (370 W). Other parameters of water quality, including total ammonia nitrogen levels, nitrite nitrogen levels, and pH values, were kept around 0.73 mg/L, 0.12 mg/L, and 7.10, respectively. The temperature of water was determined daily at 8:00, 12:00, and 18:00; the average temperature was 29.5 ± 2.3 °C. To build the determination method of HPLC, the blank samples comprising the muscle + skin, liver, plasma, gill, and kidney were collected from nine fish and saved in a freezer at −20 °C. Animal-related experimental protocols were approved by the Fish Ethics Committee of Yangtze River Fisheries Research Institute, Chinese Academy of Fishery Sciences, Wuhan, China.

Before the formal experiment, the fish were fasted for 24 h. Next, largemouth bass were administered medicated feed once a day by ad libitum administration for 5 days. The weight of medicated feed for each dose accounts for 0.5% of total body weight of fish. The final dose of EF was about 20 mg/kg/day. After 5-day administration, the sample collection was performed at time intervals of 1, 3, 5, 7, 14, 21, 28, 35, 42, and 49 days. For each sampling, ten fish were randomly captive from the barrel, and blood was collected from the caudal vessels using a 2.5 mL syringe immersed in 0.5% heparin solution. The experimenter transferred the collected blood into a 10-mL tube and separated plasma with centrifugation at 2000× *g* for 5 min. Afterwards, we also collected the muscle + skin, gill, liver, and kidney from each fish, respectively. Samples were preserved in a freezer at −20 °C up to the detection of HPLC. 

### 2.4. Sample Preparation, Equipment, and Method Validation

The sample preparation followed the method from published references [25,26]. In brief, samples of the muscle + skin, plasma, liver, gill, and kidney were unfrozen at room temperature. A total of 1 gram of tissue or 1 mL of plasma was weighed or pipetted into a 10 mL plastic tube. Next, 4 mL of acetonitrile containing 0.4% hydrochloric acid was pipetted into each tube. Samples were mixed with acidified acetonitrile for 1 min with a vortex apparatus. To dehydrate the plasma and tissue samples, 0.5 g of anhydrous magnesium sulfate was added to each tube by shaking for 1 min. The mixed samples were centrifugated at 5000× *g* for 5 min to obtain supernatant that was transferred into a new tube. According to the above-mentioned methods, the residue samples were extracted once again, and the resulting supernatants were merged. Afterwards, the extracted liquid was evaporated until dryness with high pure nitrogen (99.9%) at 45 °C using a sample concentrator. Finally, extracted substances in each tube were reconstructed in 1 mL of acetonitrile and 0.2% of formic acid water (18:82) by shaking for 1 min (200 r/min). The resulting solution was added 1 mL of n-hexane by swirling for 1 min. The mixed sample was centrifugated again at 7000× *g* for 5 min. The lower liquid was pipetted and filtrated through a 0.22 µm nylon filter for HPLC.

The equipment for determination was an Agilent HPLC system with a 1260 Infinity II (Santa Clara, CA, USA) comprising a fluorescent detector, an autosampler, a quaternary solvent manager, and a quaternary solvent pump. HPLC separated EF and CF using a column of Poroshell 120 EC-C18 (2.7 μm, 4.6 × 100 mm) at 35 °C. The flow rate of the liquid phase was 0.8 mL/min, including the aqueous phase of pure water containing 0.2% formic acid and the organic phase of pure acetonitrile. Isocratic elution was adopted in this study with a proportion of 82:18. A fluorescent detector was employed to detect target compounds with an excitation wavelength of 280 nm and an emission wavelength of 450 nm.

The method was validated in light of the guidelines of the EU Commission Decision 2021/808/EC [27]. The detailed method was cited from the published literature [1,28].

### 2.5. Statistical Analysis and Calculations

The method for WT calculation followed the guidelines of the European Medicines Agency (EMA) [29,30]. The sum of concentrations of EF and CF were lower than the limit of quantification (LOQ) and so were defined as half of LOQ. The terminal elimination of the target compound was assumed to fit a one-compartment model. The homogeneity of data was checked by the log-transformed concentration using the statistical method of Bartlett’s test and Cochran’s test. The linearity of the simulated line was checked using the F-test. Outliers were estimated by standardized residuals. If the residue value was more than +4 or less than −4, it will be determined to be an outlier. The final WT was calculated using the 95% percentile with a 95% confidence level. The calculation of WT was performed on the software WT 1.4 developed by EMA [29,30]. 

To verify the accuracy of the estimated WT, a dietary consumption risk assessment was also conducted in the study. According to concentrations of EF and CF in largemouth bass at disparate time points, EDI was estimated following the below equation [28,31].

*EDI* = *CD* × *DCAP*/*BW*

where EDI represents the estimated daily intake of EF and CF (µg/kg/d); CD indicates the level of EF and CF (µg/kg); DCAP suggests the daily consumption of edible fish tissues (kg/d), which is 0.056 kg/d cited from the Fifth China Total Diet Study; and BW represents the mean body weight of an adult (60 kg). 

The hazard quotients (HQs) of EF were estimated using the below equation [32].

*HQ* = *EDI*/*ADI*

where HQ means the hazard quotient, EDI means the estimated daily intake (µg/kg/d), and ADI is the acceptable daily intake of EF (6.2 µg/kg).

## 3. Results

### 3.1. HPLC Method Validation

In the current method, there was no peak to disturb the target compounds in blank samples, which indicates that this method had good specificity. The obtained results displayed that the limit of detection (LOD) and LOQ of EF were 0.003 and 0.01 µg/g or µg/mL in the gill, muscle + skin, liver, plasma, and kidney, respectively. The LOD and LOQ of CF were the same as that of EF in the plasma and tissues. The matrix-spiked calibration curves were constructed over concentrations from 0.01 to 2.00 and presented good linearities with R values of ≥0.9993. The related accuracy and precision values are shown in Table 1. The mean recovery rates of EF ranged from 91.51% to 119.01% in the plasma and tissues. Values of inter-day precisions ranged from 0.16% to 5.21%, and values of intra-day precisions ranged from 0.13% to 8.83%. The decision limits and detection capability ranged from 105.3 to 121.7 µg/kg or µg/L. Next, the mean recoveries of CF ranged from 83.34% to 118.15% in the plasma and tissues. Values of inter-day precisions ranged from 0.12% to 6.56%, and values of intra-day precisions ranged from 0.31% to 8.37%. The decision limits and detection capability ranged from 106.7 to 117.6 µg/kg or µg/L. During the analysis of HPLC, if levels of EF or CF in the specific plasma and tissue samples were above the upper LOQ, the related samples were attenuated with the same blank samples to repeatedly prepare and determine in line with the above-mentioned method.

### 3.2. Residue Depletion of EF and CF in Largemouth Bass

**Figure 1:**
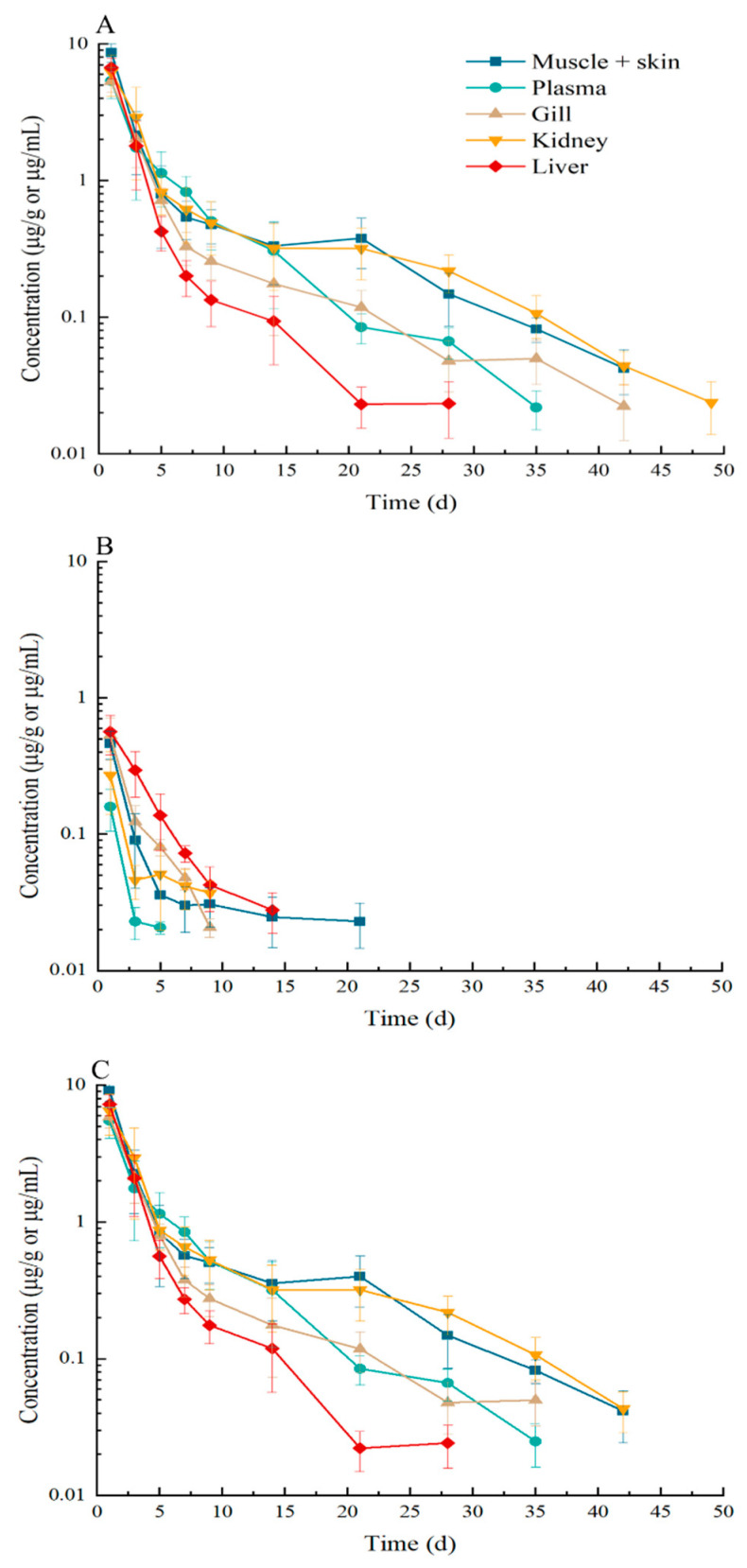
The residue depletion of enrofloxacin (**A**), ciprofloxacin (**B**), and sum of enrofloxacin and ciprofloxacin (**C**) in the muscle + skin, plasma, gill, kidney, and liver in largemouth bass (*Micropterus salmoides*) after administering enrofloxacin at a dose of 20 mg/kg for consecutive 5 days via ad libitum using medication feed.

The residue depletion profiles of EF, CF, and the sum of EF and CF in the muscle + skin, gill, kidney, liver, and plasma of largemouth bass are exhibited in Figure 1 after 5-day oral doses of EF at 20 mg/kg via medicated feed. The concentration–time curves of EF in the plasma and tissues are shown in Figure 1A. The concentrations in the plasma and tissues at the first time point were the highest than at other time points. Up to 3 days, the concentration decreased more rapidly than other later time points. Afterwards, the level of EF gradually decreased in the plasma and tissues. In the kidney and muscle + skin, the concentration of EF was higher than that in the plasma, gill, and liver. After 21 days, the terminal level of EF became the highest in the kidney. EF could be detectable up to 42, 35, 42, 49, and 28 days in the muscle + skin, plasma, gill, kidney, and liver after oral doses. The elimination half-lives were 7.13, 4.81, 6.31, 7.52, and 3.66 days in the muscle + skin, plasma, gill, kidney, and liver, respectively.

The concentration–time curves of CF in the plasma and tissues are shown in Figure 1B. The level of CF in the liver was higher than that in the plasma and other tissues. The highest concentration was also displayed at the initial time point. The related concentrations were 0.58, 0.53, 0.46, 0.27, and 0.16 µg/g or µg/mL in the liver, gill, muscle + skin, kidney, and plasma, respectively, and then the level gradually declined. The quantifiable levels in the muscle + skin, plasma, gill, kidney, and liver were until 21, 5, 9, 9, and 14 days after oral doses. The elimination half-lives were 6.26, 1.36, 1.86, 2.06, and 7.27 days, respectively.

The concentration–time curves of the sum EF and CF are shown in Figure 1C. The basic trend was similar to that of EF in the plasma and tissues. Levels in the muscle + skin and kidney were higher than in other tissues and plasma. The maximum levels were also displayed at the initial point of 1 d after oral doses, and the relevant order of concentration was muscle + skin > liver > kidney > gill > plasma, with values of 9.11, 7.26, 6.48, 5.84, and 5.49 µg/g or µg/mL, respectively. The detectable concentration could be maintained for 42, 35, 35, 42, and 28 days in the muscle + skin, plasma, gill, kidney, and liver, respectively. Up to 35 days, the levels of the sum of EF and CF were below MRL of 100 ng/g in the plasma and tissues except for the kidney. The elimination half-lives were 6.85, 4.79, 5.60, 7.27, and 3.37 days in the muscle + skin, plasma, gill, kidney, and liver, respectively.

### 3.3. The Estimation of WTs for EF in Largemouth Bass

**Figure 2:**
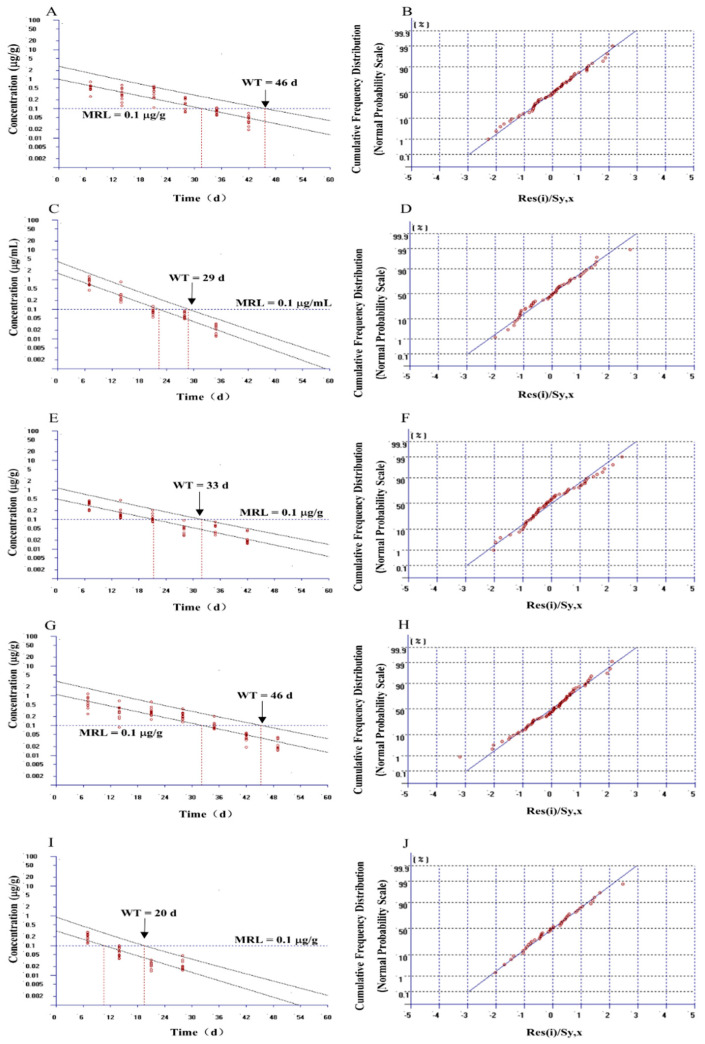
The withdrawal periods and cumulative frequency distribution of enrofloxacin and ciprofloxacin in the muscle + skin (**A**,**B**), plasma (**C**,**D**), gill (**E**,**F**), kidney (**G**,**H**), and liver (**I**,**J**) in largemouth bass (*Micropterus salmoides*) after administering enrofloxacin at a dose of 20 mg/kg for 5 consecutive days via ad libitum using medication feed.

In the present study, WT 1.4 software was employed to calculate the WTs of EF in the tissues and plasma of largemouth bass. Due to at most 7 data groups being input into the software, we selected the time points of 7, 14, 21, 28, 35, and 42 days for the muscle + skin; 7, 14, 21, 28, and 35 days for the plasma; 7, 14, 21, 28, 35, and 42 days for the gill; 7, 14, 21, 28, 35, 42, and 49 days for the kidney; and 7, 14, 21, and 28 days for the liver, respectively (Figure 2). According to the guidelines of EMA for the estimation of the WT, the sum of concentrations was less than LOQ in selected time points that were defined as half of the LOQ. The homogeneity of data was checked by the log-transformed concentration using the statistical methods of Bartlett’s test and Cochran’s test (Tables S1, S3, S5, S7 and S9). The linearity of the simulation line was inspected by the visual checking and the lack-of-fit test using F-test (Tables S2, S4, S6, S8 and S10). Outliers were assessed by standardized residuals. If the residue value is more than +4 or less than −4, it is determined to be an outlier (Figure 2). The final WT was calculated in line with China and Europe’s stipulation using the 95% percentile with a 95% confidence level. Finally, the estimated WTs were 46, 29, 33, 46, and 20 days for the muscle + skin, plasma, gill, kidney, and liver, respectively (Figure 2).

### 3.4. Risk Assessment

**Figure 3:**
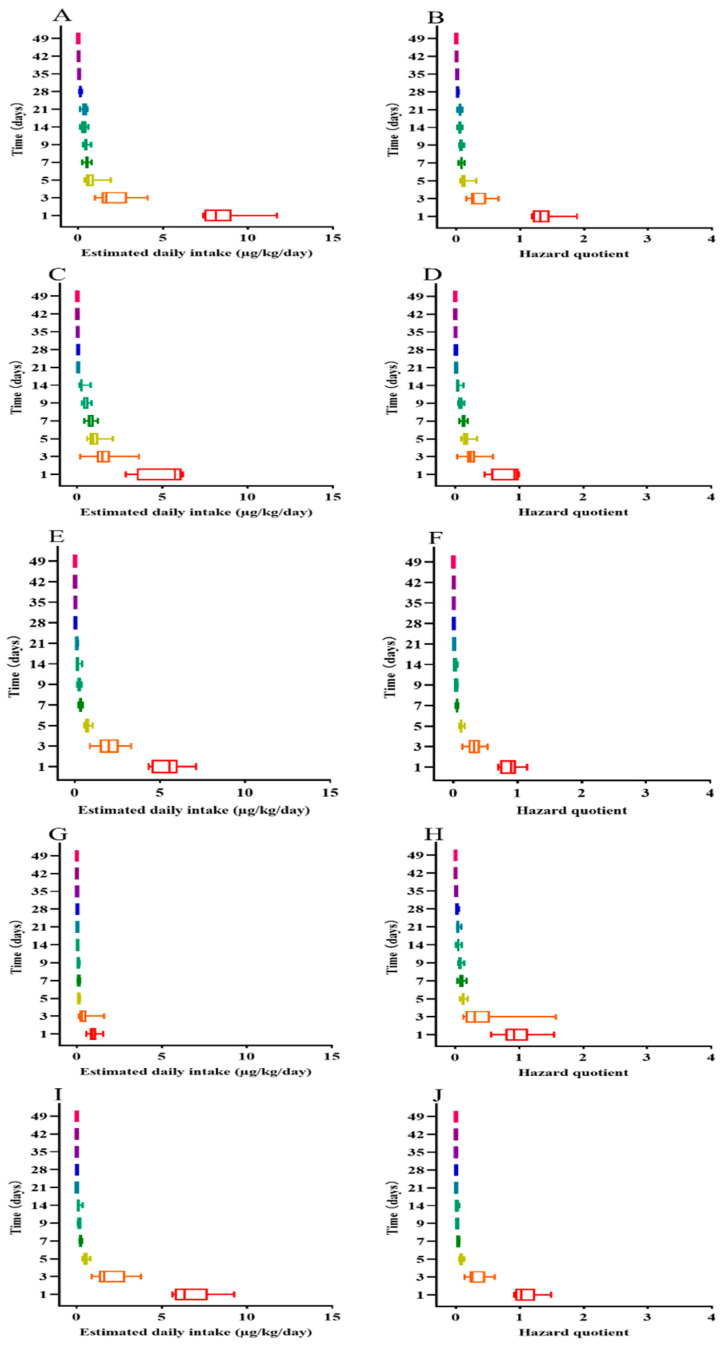
Distribution of estimated daily intakes and hazard quotients of the sum of enrofloxacin and ciprofloxacin in the muscle + skin (**A**,**B**), plasma (**C**,**D**), gill (**E**,**F**), kidney (**G**,**H**), and liver (**I**,**J**) of largemouth bass (*Micropterus salmoides*).

To verify the accuracy of the calculated WTs, the risk assessment of EF’s residues was carried out using values of EDIs and HQs. All data were used to compute the EDI and HQ values (Figure 3, Tables S11–S20). In the muscle + skin, the values of the EDI were calculated from 7.369 to 11.705, from 0.996 to 4.100, from 0.405 to 1.941, from 0.247 to 0.798, from 0.127 to 0.593, from 0.111 to 0.573, from 0.070 to 0.232, from 0.055 to 0.100, from 0.009 to 0.023, and 0.009 µg/kg/d on days 1, 3, 5, 7, 14, 21, 28, 35, 42, and 49, respectively (Figure 3A, Table S11). The values of HQ were estimated from 1.187 to 1.888, from 0.161 to 0.666, from 0.065 to 0.313, from 0.040 to 0.129, from 0.021 to 0.096, from 0.018 to 0.092, from 0.011 to 0.037, from 0.009 to 0.016, from 0.002 to 0.009, and 0.002 on the same days, respectively (Figure 3B, Table S12). The mean of HQ (1.371) on day 1 was more than 1, but the value started to be less than 1 after 3 days. On the seventh day after oral doses, the value of the EDI decreased to less than 10% of the ADI.

In the plasma, the values of the EDI were estimated from 2.848 to 6.192, from 0.189 to 3.626, from 0.575 to 2.104, from 0.432 to 1.207, from 0.169 to 0.789, from 0.051 to 0.118, from 0.045 to 0.085, from 0.009 to 0.038, 0.009, and 0.009 µg/kg/d on days 1, 3, 5, 7, 14, 21, 28, 35, 42, and 49, respectively (Figure 3C, Table S13). The values of HQ were assessed from 0.459 to 0.999, from 0.030 to 0.585, from 0.093 to 0.339, from 0.070 to 0.195, from 0.027 to 0.048, from 0.008 to 0.013, from 0.007 to 0.010, from 0.002 to 0.004, 0.002, and 0.002, respectively (Figure 3D, Table S14). The value of HQ on the first day fell below 1. Until day 9, the EDI value declined to less than 10% of the ADI.

In the gill, the values of the EDI ranged from 4.342 to 7.118, from 0.889 to 3.310, from 0.572 to 1.066, from 0.223 to 0.454, from 0.105 to 0.424, from 0.078 to 0.193, from 0.026 to 0.086, from 0.028 to 0.079, from 0.009 to 0.04, and 0.009 µg/kg/d on days 1, 3, 5, 7, 14, 21, 28, 35, 42, and 49, respectively (Figure 3E, Table S15). The values of HQ ranged from 0.700 to 1.148, from 0.143 to 0.534, from 0.092 to 0.172, from 0.036 to 0.073, from 0.017 to 0.068, from 0.013 to 0.031, from 0.004 to 0.014, from 0.004 to 0.013, from 0.002 to 0.007, and 0.002, respectively (Figure 3F, Table S16). The average value of HQ (0.879) on the first day was less than 1. Up to day 7, the values of the EDI fell below 10% of the ADI.

In the kidney, the values of the EDI were computed from 3.479 to 9.572, from 0.777 to 9.766, from 0.461 to 1.196, from 0.242 to 1.074, from 0.082 to 0.630, from 0.148 to 0.585, from 0.120 to 0.337, from 0.071 to 0.190, from 0.009 to 0.052, and from 0.009 to 0.038 µg/kg/d on days 1, 3, 5, 7, 14, 21, 28, 35, 42, and 49, respectively (Figure 3G, Table S17). The values of HQ were calculated to be from 0.561 to 1.544, from 0.125 to 1.575, from 0.074 to 0.193, from 0.039 to 0.173, from 0.013 to 0.102, from 0.024 to 0.094, from 0.019 to 0.054, from 0.011 to 0.031, from 0.002 to 0.008, and from 0.002 to 0.006, respectively (Figure 3H, Table S18). Like the plasma and gill, the mean of HQ (0.975) on the first day also fell below 1. Until day 7, the values of the EDI were also less than 10% of the ADI.

In the liver, the values of the EDI ranged from 5.630 to 9.246, from 0.889 to 3.779, from 0.345 to 0.801, from 0.184 to 0.337, from 0.054 to 0.370, from 0.009 to 0.030, from 0.009 to 0.072, 0.009, 0.009, and 0.009 µg/kg/d on days 1, 3, 5, 7, 14, 21, 28, 35, 42, and 49, respectively (Figure 3I, Table S19). The values of HQ were estimated to be from 0.908 to 1.491, from 0.143 to 0.610, from 0.056 to 0.129, from 0.030 to 0.054, from 0.009 to 0.060, from 0.002 to 0.005, from 0.002 to 0.012, 0.002, 0.002, and 0.002, respectively (Figure 3J, Table S20). Only on the first day, the mean of HQ (1.093) was more than 1. After day 3, the mean (0.314) was lower than 0.5. Up to day 5, the EDI value fell below 10% of the ADI. 

## 4. Discussion

The current study, for the first time, explored the regulation of residue depletion and the evaluation of WTs for EF in the muscle + skin, liver, gill, plasma, and kidney of largemouth bass via ad libitum administration of medication feed for 5 days in commercial fish farming based on statistical approaches. So far, oral administration via medication feed is the most important medicine administration for aquatic animals because of its many merits of low cost, easy operation, and not causing fish stress in comparison to the methods of oral gavage, injection, and medicated bath. Therefore, to obtain practical WTs of EF, the administration of ad libitum medicated feed was used to estimate WTs. Before the formal experiment, the medicated feed needed to be prepared in advance. Note that EF was added to the feed via dissolved solution. Since the realistic content of EF in the feed easily got mistaken during preparation, the feed was first smashed and dried at 40 °C for a certain time to remove the evaporative aqueous. After adding the medicine to the feed through a dissolved solution, the wet feed was also dried up to the weight being equal to the added feed and medicine. Finally, the prepared feed was sealed in a plastic bag to avoid moisture that could be absorbed into the feed to alter the proportion of medicine in the feed. 

Compared to the data from forced administration, the data from ad libitum ingestion had larger deviations and more outliers. These may be partly due to the forced administration of the medicine to aquatic animals via a bolus dose that has no loss and directly enters the fish body to reach a high concentration in a brief time. Nevertheless, through the ad libitum administration, the given amount of medicine is possibly not identical owing to a discrepancy in the food intake of each fish, even though the administered dose of EF is in line with the total body weight of the fish. The standard deviation of residue data became large, but these data were the closest to commercial fish farming. Hence, this method could correctly evaluate the WT of medicine in aquatic animals.

In China, the labelled dose of EF is 10–20 mg/kg BW for 5 or 7 days via medicated feed for aquatic animals [33]. Generally, the dose of 10 mg/kg once a day for 5 or 7 consecutive days was used extensively. However, due to the wrong administration of medicine causing medicine loss and the emergence of antimicrobial resistance, the real dose was elevated by farmers. Therefore, the residue of EF needs to be re-estimated using the highest dose in the edible tissues of fish. In the present study, the dose of 20 mg/kg was selected to evaluate the WT. According to the real medicine administration under commercial fish farming, ad libitum ingestion via medicated feed was adopted in this study. To reduce the loss of medicine and guarantee fish eating feed within 1–3 min during the medicine administration, the amount of the medicated feed only accounted for 0.5% of the total body weight of fish in the current study. This measure minimized the loss of medicated feed as soon as possible. As for the estimation of the WT for other off-labeled doses besides 20 mg/kg, we will perform a physiologically based pharmacokinetic model to obtain the WT via extrapolation. 

Since the variability of data from commercial fish farming may be larger in comparison to that from the lab experiment, the statistical model was cited to ensure the homogeneity, normality of errors, and linearity of the determined data to evaluate the WT of EF in largemouth bass. According to the guidelines of EMA, the homogeneity of data was estimated using statistical methods of Bartlett’s test and Cochran’s test. The normality of the error under the normal probability distribution was calculated using the method of ordered residuals versus their cumulative frequency distribution. Moreover, the linearity of the regression curve was assessed using the F-test and visual inspection. To calculate a proper WT, outliers need to be selected and deleted from the data group. In the current study, standardized residues were employed to confirm the outliers. In line with the standard of being below −4 or above +4, the data will be considered as the outlier. Fortunately, there are no outliers found in adopting data groups of plasma and tissues. Finally, WTs of EF in largemouth bass were calculated considering MRL of 100 µg/kg using the 95% percentile with a 95% confidence level regulated by China and EMA. Nevertheless, the Food and Drug Administration of the United States used a different method of the 99% percentile with a 95% confidence level to estimate the WT. This method may result in a longer WT at the same conditions.

After the estimation of the WT, a risk assessment was carried out for the residue levels of EF and CF to validate the safety of the WT. The harmfulness was assessed using the values of the EDI and HQ. In the liver, the computed values of HQ were above 1 in samples collected on the first day following 5-day oral doses via medicated feed at 20 mg/kg. On the third day after oral doses, the average of HQ was lower than 0.5. Until day 5, the EDI value has declined to below 10% of the ADI. In the muscle + skin, the values of HQ were also computed to be more than 1 only on the first day, and the value was below 1 from 3 days. Until day 7 after oral administrations, the values of the EDI were reduced to below 10% of the ADI. In the kidney, the values of HQ have fallen below on the first day. Until day 7, the values of the EDI were also less than 10% of the ADI. Therefore, the calculated WTs of 20, 46, and 33 days in the liver, muscle + skin, and kidney can guarantee food safety for humans.

The residues of EF and CF in the muscle + skin have a higher level compared to other tissues, especially after 14 days, suggesting EF and CF are prone to accumulate in the muscle + skin, resulting in a longer WT compared to other tissues. In previous studies, the WT was evaluated to be 45 days in the muscle + skin of striped catfish (*Pangasianodon hypophthalmus*) after daily ad libitum for five consecutive days with medicated feed at 10 mg/kg at 29.4 °C [34]. Although the dose used in the study was half that in the present study, the obtained WT of EF was similar to that in this study. This difference may be attributable to metabolic discrepancies in disparate fish species. It is reported that the WT of EF was estimated to be only 24 days in the same species of largemouth bass after a single oral gavage dose of 20 mg/kg at 28 °C [35]. This is possibly related to different administrations and medicated regimens. At a low water temperature of 12.0–13.0 °C, the WT was assessed to be 63 days in *Oncorhynchus mykiss* after the treatment with medicated feed at a dose of 10 mg/kg [36]. The reason may be that the low temperature reduces the elimination rate and metabolic rate of EF, thereby resulting in a longer WT. A previous study has proven that a decreased temperature of 1 °C could reduce 10% of the metabolic rate for fish [37]. The concentration of EF was below MRL at about 22 days in *Oreochromis niloticus* and 12 days in *Penaeus chinensis* after oral doses of 50 mg/kg via medicated feed for 7 days at 27 °C [20]. This study used a higher dose of 50 mg/kg in comparison to the present study, and the cycle of the given drug is longer than that of the current study. According to conventional logic, the estimated WT should be longer than the present study but far less than the current study. We found that the given medicated feed accounted for 2% of the total body weight of fish. Therefore, we assumed that the given medicated feed was not partly eaten by fish, resulting in a lower concentration of EF and CF. Additionally, the estimation of the WT was also conducted in the muscle + skin of *Channa argus* for 18 days after repeated oral gavage of 10 mg/kg for 5 days at 25 °C [3]. In *Exopalaemon carinicauda* receiving medicated feed at the dose of 10 mg/kg for 5 days at 18 °C, the estimated WT was 20–25 days [38]. In *Piaractus mesopotamicus* given a daily dose of 10 mg/kg via medicated feed for 10 days, the WT was estimated to be 23 days [39]. 

## 5. Conclusions

The residue depletion of EF was implemented in largemouth bass after multiple oral doses of 20 mg/kg via medicated feed in commercial fish farming. Statistical methods were cited in the study to estimate the WT. The homogeneity of data was checked using Bartlett’s test and Cochran’s test. The linearity of the regression line was checked using the lack-of-fit test via an F-test. Outliers were estimated by standardized residuals. If the residue value was more than +4 or less than −4, it was determined to be the outlier. The final WT was calculated using the 95% percentile with a 95% confidence level. Finally, WTs of EF were calculated to be 46, 29, 33, 46, and 20 days for the muscle + skin, plasma, gill, kidney, gill, and liver, respectively. After risk assessment, the values of HQ were far less than 1 after the appropriate WT.

## Figures and Tables

**Table 1:** Accuracy and precision of the method for enrofloxacin and ciprofloxacin in spiked plasma or tissues in largemouth bass (*Micropterus salmoides*) (*n* = 5).

Drug	Plasma or Tissues	Spiked Concentration (μg/g or μg/mL)	Recovery/%	Intra-Day RSD (%)	Inter-Day RSD/%
Enrofloxacin	Plasma	0.02	119.01 ± 0.41	2.14	7.18
		0.1	115.65 ± 1.21	2.56	8.83
		1	103.14 ± 1.51	2.55	2.63
	Muscle + skin	0.02	96.94 ± 2.53	3.42	3.41
		0.1	100.22 ± 0.59	2.29	2.58
		1	99.46 ± 0.8	0.4	0.32
	Gill	0.02	106.97 ± 7.28	5.21	4.65
		0.1	100.18 ± 4.11	3.23	3.09
		1	98.06 ± 0.95	0.61	0.68
	Liver	0.02	91.51 ± 0.99	3.14	6.05
		0.1	105.06 ± 1.47	3.00	2.02
		1	96.96 ± 0.3	2.21	8.01
	Kidney	0.02	112.20 ± 4.76	2.57	2.12
		0.1	102.80 ± 2.36	0.74	1.10
		1	106.79 ± 1.45	0.16	0.13
Ciprofloxacin	Plasma	0.02	93.24 ± 1.34	6.56	5.80
		0.1	84.70 ± 0.59	5.65	5.16
		1	88.55 ± 1.14	4.31	4.74
	Muscle+ skin	0.02	84.97 ± 0.63	1.83	1.94
		0.1	85.21 ± 0.85	1.87	2.23
		1	87.08 ± 1.71	0.75	0.79
	Gill	0.02	116.34 ± 3.43	5.99	8.37
		0.1	109.70 ± 2.65	5.13	4.81
		1	83.49 ± 0.47	2.40	2.06
	Liver	0.02	85.08 ± 1.84	4.37	6.00
		0.1	87.30 ± 2.07	2.08	3.51
		1	83.34 ± 1.55	4.69	6.06
	Kidney	0.02	118.15 ± 1.64	2.16	4.67
		0.1	106.14 ± 0.07	0.37	0.31
		1	91.12 ± 0.18	0.12	0.44

Note: RSD, relative standard deviation.

## Data Availability

None of the data were deposited in an official repository. The data that support the study findings are available upon request.

## Supplementary Materials

The following supporting information can be downloaded at: https://www.mdpi.com/article/10.3390/ani14162341/s1, Table S1: Statistical test for data in the muscle + skin of largemouth bass (*Micropterus salmoides*); Table S2: F-test for data in the muscle + skin of largemouth bass (*Micropterus salmoides*); Table S3: Statistical test for data in the plasma of largemouth bass (*Micropterus salmoides*); Table S4: F-test for data in the plasma of largemouth bass (*Micropterus salmoides*); Table S5: Statistical test for data in the gill of largemouth bass (*Micropterus salmoides*); Table S6: F-test for data in the gill of largemouth bass (*Micropterus salmoides*); Table S7: Statistical test for data in the kidney of largemouth bass (*Micropterus salmoides*); Table S8: F-test for data in the kidney of largemouth bass (*Micropterus salmoides*); Table S9: Statistical test for data in the liver of largemouth bass (*Micropterus salmoides*); Table S10: F-test for data in the liver of largemouth bass (*Micropterus salmoides*); Table S11: The estimated daily intake of enrofloxacin and ciprofloxacin in the muscle + skin of largemouth bass (*Micropterus salmoides*) via food consumption (µg/kg/day); Table S12: The hazard quotient of enrofloxacin and ciprofloxacin in the muscle + skin of largemouth bass (*Micropterus salmoides*) via food consumption; Table S13: The estimated daily intake of enrofloxacin and ciprofloxacin in the plasma of largemouth bass (*Micropterus salmoides*) via food consumption (µg/kg/day); Table S14: The hazard quotient of enrofloxacin and ciprofloxacin in the plasma of largemouth bass (*Micropterus salmoides*) via food consumption; Table S15: The estimated daily intake of enrofloxacin and ciprofloxacin in the gill of largemouth bass (*Micropterus salmoides*) via food consumption (µg/kg/day); Table S16: The hazard quotient of enrofloxacin and ciprofloxacin in the gill of largemouth bass (*Micropterus salmoides*) via food consumption; Table S17: The estimated daily intake of enrofloxacin and ciprofloxacin in the kidney of largemouth bass (*Micropterus salmoides*) via food consumption (µg/kg/day); Table S18: The hazard quotient of enrofloxacin and ciprofloxacin in the kidney of largemouth bass (*Micropterus salmoides*) via food consumption; Table S19: The estimated daily intake of enrofloxacin and ciprofloxacin in the liver of largemouth bass (*Micropterus salmoides*) via food consumption (µg/kg/day); Table S20: The hazard quotient of enrofloxacin and ciprofloxacin in the liver of largemouth bass (*Micropterus salmoides*) via food consumption.

## Abbreviations

CFD, cumulative frequency distribution; CF, ciprofloxacin; EF, enrofloxacin; EDI, estimated daily intake; EMA, European Medicines Agency; HPLC, high-performance liquid chromatography; HQ, hazard quotient; LOD, limit of detection; LOQ, limit of quantification; MRL, maximum residue limit; RSD, relative standard deviation; WT, withdrawal time.
