# Supplementary material for: A Modified Calculation of the Withdrawal Time and a Risk Assessment of Enrofloxacin in Micropterus salmoides after Its Ad Libitum Administration via Medicated Feed in the Commercial Aquaculture
Source: Animals (Basel). 2024 Aug 14;14(16):2341. doi: 10.3390/ani14162341 (PMC11350749; doi:10.3390/ani14162341)
Supplement: Supplementary file 1 [file animals-14-02341-s001.zip › animals-3127413-supplementary.pdf]

Supplementary materials

A modified calculation of withdrawal time and risk assessment for enrofloxacin in *Micropterus salmoides* after *ad libitum* via medicated feed in natural fish farming

Ning Xu[1], Yongzhen Ding[2]*, Xiaohui Ai[1]*

[1] Yangtze River Fisheries Research Institute, Chinese Academy of Fishery Sciences, Wuhan 430223, China

[2] Agro-Environmental Protection Institute, Ministry of Agriculture and Rural Affairs, Tianjin 300191, China

Correspondence to:

Xiaohui Ai, Yangtze River Fisheries Research Institute, Chinese Academy of Fishery Sciences, No. 8 Wuda Park Road 1, Wuhan 430223, Hubei Province, China. E-mail: aixh@yfi.ac.cn.

Yongzhen Ding, Agro-Environmental Protection Institute, Ministry of Agriculture and Rural Affairs, No. 31 Fukang Road, Tianjin, 300191, China. Email: dingyongzhen@caas.cn.

Contents

Supplementary tables 3
Table S1 3
Table S2 3
Table S3 4
Table S4 4
Table S5 5
Table S6 5
Table S7 6
Table S8 6
Table S9 7
Table S10 7
Table S11 8
Table S12 8
Table S13 9
Table S14 9
Table S15 10
Table S16 10
Table S17 11
Table S18 11
Table S19 12
Table S20 12

Supplementary tables

Table S1. Statistical test for data in muscle + skin of largemouth bass (*Micropterus salmoides*)

Statistical test	Test value	Degrees of freedom	Probability	Significance
Cochran's test	0.26	df1 = 6<br>df2 = 9	$P > 0.05$	NS
Bartlett's test	7.80	Df = 5	$P > 0.05$	NS

Note: NS, no significant.

Table S2. F-test for data in muscle + skin of largemouth bass (*Micropterus salmoides*)

Source of variation	Test value	Degrees of freedom	Probability	Significance
Between-group means and the regression line	4.46	4	$P > 0.05$	NS
Within groups (departure of y-values from their group mean)		54		

Note: NS, no significant.

Table S3. Statistical test for data in plasma of largemouth bass (*Micropterus salmoides*)

Statistical test	Test value	Degrees of freedom	Probability	Significance
Cochran's test	0.35	df1 = 5<br>df2 = 9	*P* > 0.05	NS
Bartlett's test	4.78	Df = 4	*P* > 0.05	NS

Note: NS, no significant.

Table S4. F-test for data in hepatopancreas

Source of variation	Test value	Degrees of freedom	Probability	Significance
Between-group means and the regression line	6.80	3	*P* > 0.05	NS
Within groups (departure of y-values from their group mean)		45		

Note: NS, no significant.

Table S5. Statistical test for data in gill of largemouth bass (*Micropterus salmoides*)

Statistical test	Test value	Degrees of freedom	Probability	Significance
Cochran's test	0.23	df1 = 6<br>df2 = 9	$P > 0.05$	NS
Bartlett's test	1.90	Df = 5	$P > 0.05$	NS

Note: NS, no significant; NA, not available.

Table S6. F-test for data in gill of largemouth bass (*Micropterus salmoides*)

Source of variation	Test value	Degrees of freedom	Probability	Significance
Between-group means and the regression line	3.76	4	$P > 0.05$	NS
Within groups (departure of y-values from their group mean)		54		

Note: NS, no significant.

Table S7. Statistical test for data in kidney of largemouth bass (*Micropterus salmoides*)

Statistical test	Test value	Degrees of freedom	Probability	Significance
Cochran's test	0.29	df1 = 7<br>df2 = 9	*P* > 0.05	NS
Bartlett's test	6.27	Df = 6	*P* > 0.05	NS

Note: NS, no significant.

Table S8. F-test for data in kidney of largemouth bass (*Micropterus salmoides*)

Source of variation	Test value	Degrees of freedom	Probability	Significance
Between-group means and the regression line	6.55	5	*P* > 0.05	NS
Within groups (departure of y-values from their group mean)		63		

Note: NS, no significant.

Table S9. Statistical test for data in liver of largemouth bass (*Micropterus salmoides*)

Statistical test	Test value	Degrees of freedom	Probability	Significance
Cochran's test	0.32	df1 = 4<br>df2 = 9	$P > 0.05$	NS
Bartlett's test	1.19	Df = 3	$P > 0.05$	NS

Note: NS, no significant.

Table S10. F-test for data in kidney of largemouth bass (*Micropterus salmoides*)

Source of variation	Test value	Degrees of freedom	Probability	Significance
Between-group means and the regression line	17.14	2	$P > 0.05$	NS
Within groups (departure of y-values from their group mean)		36		

Note: NS, no significant.

Table S11. The estimated daily intake of enrofloxacin and ciprofloxacin in muscle + skin of largemouth bass (*Micropterus salmoides*) via food consumption (μg/kg/day)

Animal number	1 d	3 d	5 d	7 d	14 d	21 d	28 d	35 d	42 d	49 d
1	8.462	1.863	0.857	0.788	0.518	0.509	0.141	0.086	0.032	0.009*
2	8.065	3.442	0.478	0.443	0.127	0.377	0.133	0.093	0.050	0.009*
3	8.196	1.437	0.405	0.568	0.593	0.522	0.076	0.061	0.031	0.009*
4	9.507	0.996	0.411	0.247	0.254	0.403	0.070	0.078	0.023	0.009*
5	7.369	4.100	0.605	0.600	0.291	0.226	0.143	0.059	0.064	0.009*
6	11.705	1.453	0.492	0.798	0.481	0.242	0.214	0.069	0.009*	0.009*
7	8.002	2.145	1.941	0.469	0.328	0.299	0.092	0.055	0.053	0.009*
8	8.896	2.709	0.650	0.444	0.161	0.111	0.189	0.092	0.043	0.009*
9	7.443	1.346	1.024	0.412	0.205	0.471	0.232	0.074	0.035	0.009*
10	7.360	1.424	0.896	0.527	0.359	0.573	0.090	0.100	0.046	0.009*

Note: *represents concentration values of enrofloxacin and ciprofloxacin below the limit of quantitation (LOQ) that were set to 1/2 LOQ.

Table S12. The hazard quotient of enrofloxacin and ciprofloxacin in muscle + skin of largemouth bass (*Micropterus salmoides*) via food consumption

Animal number	1 d	3 d	5 d	7 d	9 d	14 d	21 d	28 d	35 d	42 d	49 d
1	1.365	0.300	0.138	0.127	0.065	0.084	0.082	0.023	0.014	0.005	0.002
2	1.301	0.555	0.077	0.071	0.060	0.021	0.061	0.021	0.015	0.008	0.002
3	1.322	0.232	0.065	0.092	0.045	0.096	0.084	0.012	0.010	0.005	0.002
4	1.533	0.161	0.066	0.040	0.086	0.041	0.065	0.011	0.013	0.004	0.002
5	1.189	0.661	0.098	0.097	0.103	0.047	0.036	0.023	0.010	0.010	0.002
6	1.888	0.234	0.079	0.129	0.121	0.078	0.039	0.035	0.011	0.002	0.002
7	1.291	0.346	0.313	0.076	0.071	0.053	0.048	0.015	0.009	0.009	0.002
8	1.435	0.437	0.105	0.072	0.068	0.026	0.018	0.030	0.015	0.007	0.002
9	1.200	0.217	0.165	0.066	0.069	0.033	0.076	0.037	0.012	0.006	0.002
10	1.187	0.230	0.145	0.085	0.070	0.058	0.092	0.014	0.016	0.007	0.002

Table S13. The estimated daily intake of enrofloxacin and ciprofloxacin in plasma of largemouth bass (*Micropterus salmoides*) via food consumption (μg/kg/day)

Animal number	1 d	3 d	5 d	7 d	14 d	21 d	28 d	35 d	42 d	49 d
1	3.368	1.477	1.093	0.432	0.222	0.073	0.085	0.027	0.009*	0.009*
2	6.137	1.158	1.148	0.851	0.169	0.069	0.079	0.009*	0.009*	0.009*
3	6.155	1.501	0.931	1.207	0.305	0.118	0.049	0.031	0.009*	0.009*
4	6.192	1.085	0.575	0.955	0.211	0.098	0.084	0.009*	0.009*	0.009*
5	5.725	1.259	2.104	0.813	0.261	0.086	0.046	0.038	0.009*	0.009*
6	5.919	3.626	0.692	0.664	0.210	0.051	0.050	0.020	0.009*	0.009*
7	5.767	0.189	0.760	1.025	0.301	0.086	0.045	0.036	0.009*	0.009*
8	3.531	2.871	0.852	0.668	0.193	0.070	0.059	0.009*	0.009*	0.009*
9	2.848	1.627	0.928	0.645	0.295	0.061	0.071	0.023	0.009*	0.009*
10	5.638	1.621	1.610	0.619	0.798	0.077	0.053	0.029	0.009*	0.009*

Note: *represents concentration values of enrofloxacin and ciprofloxacin below the limit of quantitation (LOQ) that were set to 1/2 LOQ.

Table S14. The hazard quotient of enrofloxacin and ciprofloxacin in plasma of largemouth bass (*Micropterus salmoides*) via food consumption

Animal number	1 d	3 d	5 d	7 d	14 d	21 d	28 d	35 d	42 d	49 d
1	0.543	0.238	0.176	0.070	0.036	0.012	0.014	0.004	0.002	0.002
2	0.990	0.187	0.185	0.137	0.027	0.011	0.013	0.002	0.002	0.002
3	0.993	0.242	0.150	0.195	0.049	0.019	0.008	0.005	0.002	0.002
4	0.999	0.175	0.093	0.154	0.034	0.016	0.013	0.002	0.002	0.002
5	0.923	0.203	0.339	0.131	0.042	0.014	0.007	0.006	0.002	0.002
6	0.955	0.585	0.112	0.107	0.034	0.008	0.008	0.003	0.002	0.002
7	0.930	0.030	0.123	0.165	0.049	0.014	0.007	0.006	0.002	0.002
8	0.570	0.463	0.137	0.108	0.031	0.011	0.010	0.002	0.002	0.002
9	0.459	0.262	0.150	0.104	0.048	0.010	0.011	0.004	0.002	0.002
10	0.909	0.261	0.260	0.100	0.129	0.012	0.009	0.005	0.002	0.002

Table S15. The estimated daily intake of enrofloxacin and ciprofloxacin in gill of largemouth bass (*Micropterus salmoides*) via food consumption (μg/kg/day)

Animal number	1 d	3 d	5 d	7 d	14 d	21 d	28 d	35 d	42 d	49 d
1	4.342	2.511	0.722	0.254	0.130	0.078	0.033	0.036	0.009*	0.009*
2	6.043	2.001	0.644	0.223	0.135	0.152	0.051	0.035	0.009*	0.009*
3	7.118	1.655	0.776	0.239	0.126	0.193	0.052	0.079	0.019	0.009*
4	4.881	1.281	0.752	0.342	0.108	0.098	0.029	0.032	0.009*	0.009*
5	4.505	2.755	1.066	0.438	0.138	0.100	0.052	0.032	0.037	0.009*
6	5.232	1.979	0.595	0.454	0.105	0.093	0.048	0.028	0.009*	0.009*
7	6.079	1.523	0.609	0.395	0.198	0.096	0.026	0.054	0.009*	0.009*
8	4.482	2.090	0.572	0.358	0.111	0.081	0.027	0.055	0.041	0.009*
9	5.881	0.889	0.816	0.429	0.162	0.090	0.044	0.060	0.020	0.009*
10	5.952	3.310	0.872	0.379	0.424	0.119	0.086	0.054	0.009*	0.009*

Note:*represents concentration values of enrofloxacin and ciprofloxacin below the limit of quantitation (LOQ) that were set to 1/2 LOQ.

Table S16. The hazard quotient of enrofloxacin and ciprofloxacin in gill of largemouth bass (*Micropterus salmoides*) via food consumption

Animal number	1 d	3 d	5 d	7 d	14 d	21 d	28 d	35 d	42 d	49 d
1	0.700	0.405	0.116	0.041	0.021	0.013	0.005	0.006	0.002	0.002
2	0.975	0.323	0.104	0.036	0.022	0.024	0.008	0.006	0.002	0.002
3	1.148	0.267	0.125	0.038	0.020	0.031	0.008	0.013	0.003	0.002
4	0.787	0.207	0.121	0.055	0.017	0.016	0.005	0.005	0.002	0.002
5	0.727	0.444	0.172	0.071	0.022	0.016	0.008	0.005	0.006	0.002
6	0.844	0.319	0.096	0.073	0.017	0.015	0.008	0.004	0.002	0.002
7	0.981	0.246	0.098	0.064	0.032	0.016	0.004	0.009	0.002	0.002
8	0.723	0.337	0.092	0.058	0.018	0.013	0.004	0.009	0.007	0.002
9	0.949	0.143	0.132	0.069	0.026	0.015	0.007	0.010	0.003	0.002
10	0.960	0.534	0.141	0.061	0.068	0.019	0.014	0.009	0.002	0.002

Table S17. The estimated daily intake of enrofloxacin and ciprofloxacin in kidney of largemouth bass (*Micropterus salmoides*) via food consumption (μg/kg/day)

Animal number	1 d	3 d	5 d	7 d	14 d	21 d	28 d	35 d	42 d	49 d
1	9.290	3.118	0.519	0.242	0.290	0.148	0.233	0.110	0.009*	0.009*
2	5.252	2.143	0.645	0.554	0.182	0.279	0.192	0.074	0.051	0.033
3	3.605	1.028	0.876	0.481	0.269	0.391	0.153	0.190	0.034	0.020
4	6.125	4.116	0.461	0.510	0.163	0.247	0.184	0.103	0.052	0.009
5	5.600	0.777	1.196	0.631	0.082	0.199	0.337	0.090	0.044	0.034
6	9.572	1.653	0.850	0.818	0.381	0.225	0.247	0.079	0.030	0.009*
7	5.777	9.766	0.785	0.536	0.381	0.266	0.147	0.097	0.045	0.038
8	6.332	0.866	0.840	0.450	0.356	0.585	0.120	0.083	0.037	0.009*
9	3.479	2.945	0.805	1.074	0.249	0.340	0.242	0.098	0.052	0.009*
10	5.399	1.115	1.137	0.839	0.630	0.292	0.179	0.071	0.046	0.009*

Note:*represents concentration values of enrofloxacin and ciprofloxacin below the limit of quantitation (LOQ) that were set to 1/2 LOQ.

Table S18. The hazard quotient of enrofloxacin and ciprofloxacin in kidney of largemouth bass (*Micropterus salmoides*) via food consumption

Animal number	1 d	3 d	5 d	7 d	14 d	21 d	28 d	35 d	42 d	49 d
1	1.498	0.503	0.084	0.039	0.047	0.024	0.038	0.018	0.002	0.002
2	0.847	0.346	0.104	0.089	0.029	0.045	0.031	0.012	0.008	0.005
3	0.582	0.166	0.141	0.078	0.043	0.063	0.025	0.031	0.005	0.003
4	0.988	0.664	0.074	0.082	0.026	0.040	0.030	0.017	0.008	0.002
5	0.903	0.125	0.193	0.102	0.013	0.032	0.054	0.014	0.007	0.005
6	1.544	0.267	0.137	0.132	0.061	0.036	0.040	0.013	0.005	0.002
7	0.932	1.575	0.127	0.086	0.062	0.043	0.024	0.016	0.007	0.006
8	1.021	0.140	0.135	0.073	0.057	0.094	0.019	0.013	0.006	0.002
9	0.561	0.475	0.130	0.173	0.040	0.055	0.039	0.016	0.008	0.002
10	0.871	0.180	0.183	0.135	0.102	0.047	0.029	0.011	0.007	0.002

Table S19. The estimated daily intake of enrofloxacin and ciprofloxacin in liver of largemouth bass (*Micropterus salmoides*) via food consumption (μg/kg/day)

Animal number	1 d	3 d	5 d	7 d	14 d	21 d	28 d	35 d	42 d	49 d
1	5.630	1.380	0.622	0.193	0.069	0.020	0.009*	0.009*	0.009*	0.009*
2	7.854	1.557	0.414	0.184	0.054	0.022	0.009*	0.009*	0.009*	0.009*
3	5.669	3.779	0.499	0.232	0.065	0.025	0.019	0.009*	0.009*	0.009*
4	7.100	2.832	0.345	0.191	0.060	0.023	0.009*	0.009*	0.009*	0.009*
5	6.452	0.889	0.776	0.337	0.072	0.009	0.030	0.009*	0.009*	0.009*
6	9.246	1.431	0.373	0.314	0.102	0.009	0.009*	0.009*	0.009*	0.009*
7	5.788	1.820	0.396	0.285	0.129	0.030	0.009*	0.009*	0.009*	0.009*
8	6.263	2.934	0.452	0.232	0.058	0.026	0.009*	0.009*	0.009*	0.009*
9	6.130	1.175	0.553	0.285	0.127	0.020	0.049	0.009*	0.009*	0.009*
10	7.647	1.688	0.801	0.287	0.370	0.022	0.072	0.009*	0.009*	0.009*

Note: *represents concentration values of enrofloxacin and ciprofloxacin below the limit of quantitation (LOQ) that were set to 1/2 LOQ.

Table S20. The hazard quotient of enrofloxacin and ciprofloxacin in liver of largemouth bass (*Micropterus salmoides*) via food consumption

Animal number	1 d	3 d	5 d	7 d	14 d	21 d	28 d	35 d	42 d	49 d
1	0.908	0.223	0.100	0.031	0.011	0.003	0.002	0.002	0.002	0.002
2	1.267	0.251	0.067	0.030	0.009	0.004	0.002	0.002	0.002	0.002
3	0.914	0.610	0.081	0.037	0.011	0.004	0.003	0.002	0.002	0.002
4	1.145	0.457	0.056	0.031	0.010	0.004	0.002	0.002	0.002	0.002
5	1.041	0.143	0.125	0.054	0.012	0.002	0.005	0.002	0.002	0.002
6	1.491	0.231	0.060	0.051	0.016	0.002	0.002	0.002	0.002	0.002
7	0.934	0.293	0.064	0.046	0.021	0.005	0.002	0.002	0.002	0.002
8	1.010	0.473	0.073	0.037	0.009	0.004	0.002	0.002	0.002	0.002
9	0.989	0.190	0.089	0.046	0.021	0.003	0.008	0.002	0.002	0.002
10	1.233	0.272	0.129	0.046	0.060	0.004	0.012	0.002	0.002	0.002
